# Tackling the challenges of bioimage analysis

**DOI:** 10.7554/eLife.64384

**Published:** 2020-12-02

**Authors:** Daniël M Pelt

**Affiliations:** Leiden Institute of Advanced Computer Science, Leiden UniversityLeidenNetherlands

**Keywords:** bioimage informatics, deep learning, reproducibility, objectivity, validity, fluorescence microscopy, Mouse, Zebrafish

## Abstract

Using multiple human annotators and ensembles of trained networks can improve the performance of deep-learning methods in research.

**Related research article** Segebarth D, Griebel M, Stein N, R von Collenberg C, Martin C, Fiedler D, Comeras LB, Sah A, Schoeffler V, Lüffe T, Dürr A, Gupta R, Sasi M, Lillesaar C, Lange MD, Tasan RO, Singewald N, Pape HC, Flath CM, Blum R. 2020. On the objectivity, reliability, and validity of deep learning enabled bioimage analyses. *eLife* **9**:e59780. doi: 10.7554/eLife.59780

Deep learning has shown promising results in a wide range of imaging problems in recent years (LeCun et al., 2015), and has the potential to help researchers by automating the analysis of various kinds of biological images (Ronneberger et al., 2015). However, many deep-learning methods require a large amount of 'training data' in order to produce useful results, and this is often not available for bioimage analysis. There is, therefore, a need for deep-learning methods that can make the most from a limited amount of training data. Now, in eLife, Robert Blum (University Hospital Würzburg), Christoph Flath (University of Würzburg) and colleagues – including Dennis Segebarth and Matthias Griebel as joint first authors – provide guidance on how to do this in bioimage analysis (Segebarth et al., 2020).

A common approach to applying deep learning to image analysis involves 'convolutional neural networks': these networks take an input image (such as a microscopy image) and perform many mathematical operations on it to produce an output image (such as a corresponding image with interesting features annotated). A convolutional neural network is characterized by a set of 'learnable parameters', which have to be set to the correct values for the network to perform a given task. The act of finding the correct values for these parameters is called 'training', and several different training techniques are used in practice.

In supervised learning, training is performed using a set of input images and target output images, and the learnable parameters are iteratively adjusted until the output images produced by the network match the target images. It is important to note that supervised learning involves a large amount of randomness, and that training multiple networks using the same data will result in different networks that produce (slightly) different output images.

In bioimage analysis, a common task is to annotate certain structures in images produced by techniques such as microscopy, cryo-EM or X-ray tomography (Meijering et al., 2016). However, the complicated nature of biological images means that this annotation often has to be done by a human expert, which is time-consuming, labour-intensive and subjective (Figure 1A). Supervised deep learning could provide a way to automate the annotation process, reducing the burden on human experts and enabling analysis of a significantly larger set of images. However, annotating the input images needed to train the network also requires a significant amount of time and effort from a human expert.

**Figure 1.**
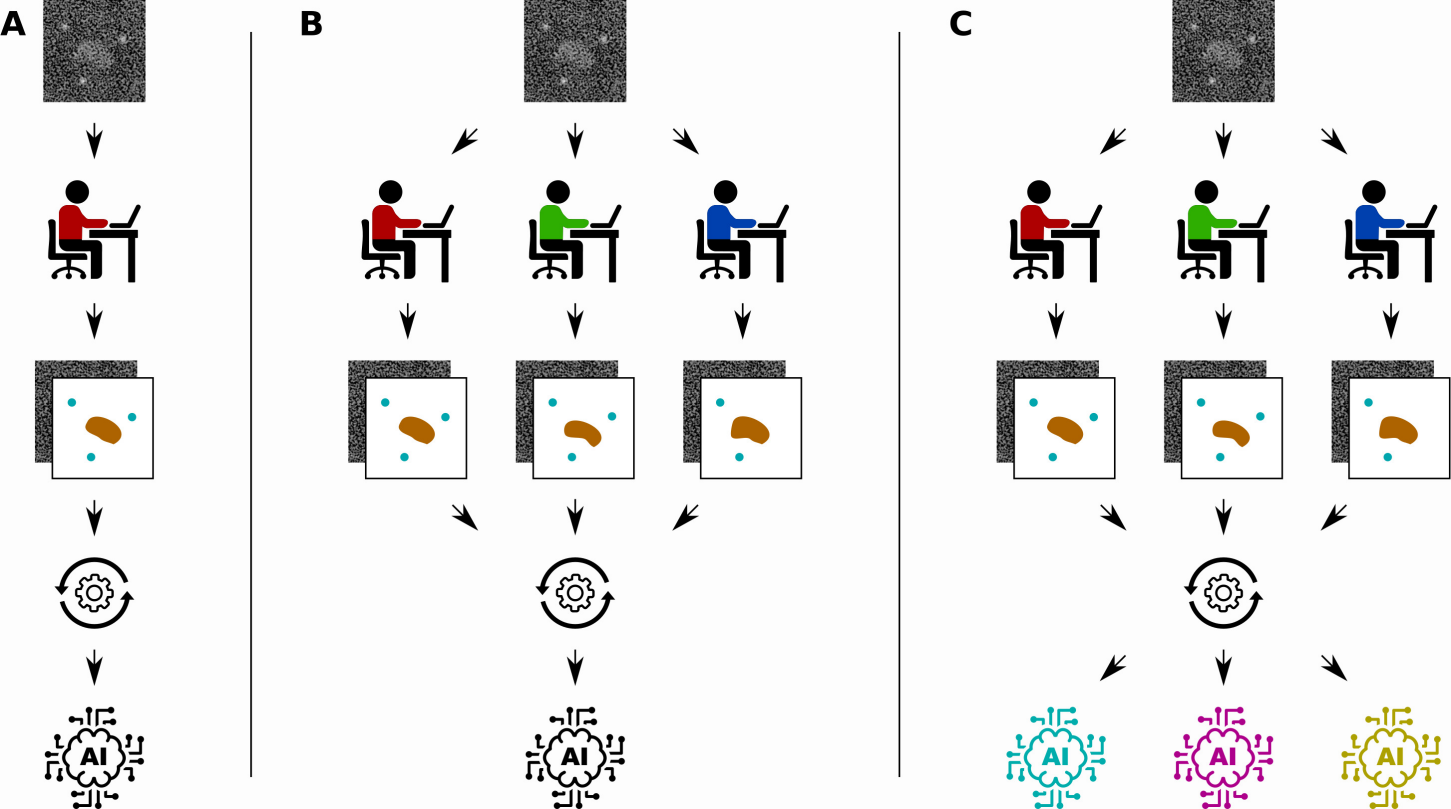
Different ways to train a convolutional neural network. Segebarth et al. compare three techniques for training convolutional neural networks to analyze bioimages. (**A**) In the standard approach a single human expert annotates images for training a single network. (**B**) In a second approach multiple human experts annotate the same images, and consensus images are used for training: this improves the objectivity of the trained network. (**C**) In a third approach, a technique called model ensembling is added to the second approach, meaning that multiple networks are trained with the same consensus images: this improves the reliability of the results.

Several properties of a trained network are important for real-world applications. The first is its objectivity, referring to a lack of influences from the subjective nature of human annotations. The second is its reliability, meaning that the trained network should consistently annotate similar features in the same way. The third is its validity, referring to the truthfulness of the network output (that is, did we annotate what we intended to?). In practice, it is often the case that objectivity, reliability and validity are difficult to achieve when the amount of training data is limited.

Various approaches to improve the objectivity, reliability and validity of convolutional neural networks have been proposed. Some involve adapting the structure of the network themselves by, for example, reducing the number of learnable parameters (Pelt and Sethian, 2018), and some involve adapting the training method by, for example, randomly ignoring parts of the network during training (Srivastava et al., 2014). A different approach is to focus on the training data used in supervised learning. Given a network structure and a training method, how can the training data set be optimized to improve objectivity, reliability and validity? In other words, given the time-consuming, labour-intensive and subjective nature of manual annotation, how can a limited period of time from human experts be best utilized to produce a training data set? These questions are currently the subject of active research.

Segebarth et al. investigate two techniques for improving the objectivity, reliability and validity of trained convolutional neural networks in bioimage analysis. First, they investigate the use of multiple human experts to annotate the same set of training images (Figure 1B). The different annotations of each input image are then combined to create a *consensus* target output image. Since each human expert has their own intended and unintended biases, networks that are trained with data from a single human expert might include the biases of the expert. Using consensus images from multiple experts during training can improve the objectivity of the resulting networks by removing these biases from the training data.

The second technique is to train multiple convolutional neural networks using the same training data set, and then combine the results when the networks are used to analyse new images (Figure 1C). This technique, called model ensembling, has already proven successful in a wide range of applications (Krizhevsky et al., 2017). Model ensembling is based on the randomness involved in training described above: because of this randomness, each trained network will be implicitly biased in their results. By combining the output of multiple networks, these biases are effectively removed, resulting in more reliable results.

A key contribution of Segebarth et al. was to perform extensive experiments on real images and show that the use of consensus images and model ensembles does indeed improve objectivity, reliability and validity. This provides a recipe for optimizing the generation of training data and for making efficient use of the available data, although this recipe still requires a significant amount of human expert time since each image has to be annotated by multiple experts. The results could also help researchers trying to understand how biases affect trained networks, which could lead to improved network structures and training approaches (Müller et al., 2019). And although many questions and challenges remain, the work of Segebarth et al. represents an important step forward in the effort to make the use of deep learning in bioimage analysis feasible.
